# Research on the Mechanical and Physical Properties of Basalt Fiber-Reinforced Pervious Concrete

**DOI:** 10.3390/ma15196527

**Published:** 2022-09-20

**Authors:** Jian Wu, Qian Pang, Yuanyuan Lv, Jinpeng Zhang, Shan Gao

**Affiliations:** 1 Shaanxi Key Laboratory of Safety and Durability of Concrete Structures, Xijing University, Xi’an 710123, China; 2 The Youth Innovation Teen of Shaanxi Universities, Xijing University, Xi’an 710123, China; 3 School of Civil Engineering, Harbin Institute of Technology, Harbin 150090, China

**Keywords:** pervious concrete, basalt fiber, strength, permeability, mesostructure

## Abstract

The aim of this study was to investigate the properties of fiber-reinforced pervious concrete. Ordinary cement, silica fume, coarse aggregate, and basalt fibers were used to produce the concrete mix. The fibers were mixed with pervious concrete at the levels of 0 kg/m^3^, 2 kg/m^3^, 4 kg/m^3^, 6 kg/m^3^, and 8 kg/m^3^ to the investigate their influence on the mechanical and physical properties of pervious concrete. It could be observed that the cubic compressive strength, axial compressive strength, and flexural strength increased and then decreased as the content of basalt fiber increased, while the permeability and porosity of the pervious concrete decreased with the increase in the basalt fiber content. The mesostructure of pervious concrete was also studied through industrial computed tomography (ICT); the testing phenomenon showed that the fibers had a significant influence on the arrangement of the aggregate, cement paste, and the interfacial transition zone, and excessive basalt fiber resulted in poor characteristics of the interfacial transition zone (ITZ) and inferior strength properties. It was found that incorporating a basalt fiber content of 4 kg/m^3^ could achieve a balance between the mechanical and physical properties of pervious concrete, which was suitable for structural applications.

## 1. Introduction

Nowadays, various types of concrete gradually appear with the development of the economy, such as pervious concrete, fiber-reinforced concrete, and self-healing concrete [1,2,3,4]. Among these new types of concrete, pervious concrete has gradually become a research hotspot. Pervious concrete is a material consisting of cement, water, and coarse aggregates, which could protect the environment by replenishing the groundwater and reducing storm water runoff [1,5]. This could help to maintain ecological balance and alleviate the urban heat island effect. Therefore, although the construction cost has increased, pervious concrete is widely used all over the world in road paving, sidewalks, and pathways due to its high porosity and good thermal properties [6]. However, in order to obtain high porosity and permeability, pervious concrete often contains little or no fine aggregate [7], which results in an obvious reduction in strength and durability. Therefore, although pervious concrete can better promote the protection of the environment and the management of rainwater, its application is limited due to low strength [8]. Based on the previous research, the parameters that influence the properties are the cement content, water-to-cement, and the ratio of cement-to-aggregate [9,10,11].

In order to protect the environment, recycled aggregate is gradually being introduced as a partial replacement for natural coarse aggregate [12,13,14]. The reason for this is that a huge amount of concrete waste is produced every year, which not only requires much space for its disposal, but also pollutes the environment [15]. Although the addition of recycled aggregate can increase the permeability, due to its defects, excessive recycled aggregate will adversely affect the mechanical properties [16,17,18]. Kachouh et al. [19] found that the optimum recycled aggregate replacement ratio was 30%, while the negative effect on the compressive strength could be seen when the recycled aggregate content was higher. Yap et al. [17] showed that the compressive strength of pervious concrete with 60–100% recycled aggregate as a partial replacement for natural aggregate was 20–40% lower than that of the pervious concrete with 100% natural aggregate. The study of El-Hassan [20] showed that the compressive strength of samples with 100% recycled aggregate was 78% lower than the control group. Given these deficiencies, recycled aggregate was mainly used in low-to-medium strength concrete and pavement applications [21,22]. Therefore, natural aggregate is used in this paper to improve the mechanical performance of pervious concrete.

Supplementary cementitious materials could improve the mechanical and physical properties of concrete due to the effects of microfilling and pozzolanic reactivity [23,24]. Amongst these materials, silica fume is more commonly used because it could refine the microstructure (pores and gaps) of the concrete matrix, and also produce additional gel due to the fact that the reactive silica reacts with the Ca(OH)_2_ of the cement [25]. Chen et al. [26] studied the influence of silica fume on the strength, fracture, and fatigue of pervious concrete. The results showed that replacing the cement with silica fume could enhance the bonding effect between the aggregate and cement paste. Adil et al. [27] found that the mechanical properties of pervious concrete could be improved with a 5% content of silica fume, while the silica fume was not affected by the addition of silica fume. Amornsrivilai et al. [28] observed that the microstructure of pervious concrete could be improved due to the pore refinement by silica fume. Therefore, silica fume is added into the concrete mix to enhance the mechanical and physical properties of pervious concrete.

Besides the silica fume, another technique that is being widely used is fiber reinforcement. The reinforcing fibers could not only improve the seismic behavior of the structure [29,30], but also enhance the mechanical performance of concrete due to the bridging action of the fibers [31,32,33]. Industrial fibers have been successfully added into concrete to improve the mechanical properties, especially the tensile flexural strength. Basalt fibers are widely used in the production of fiber-reinforced concrete due to their low cost, simple production process, and high elastic modulus [34,35]. Mahapara et al. [36] concluded that the mechanical strength of basalt fiber-reinforced concrete increased by 88%, 89%, and 92% at 7 days, 28 days, and 90 days, respectively. Abbass et al. [36] found that concrete with an appropriate basalt fiber content could reduce the weight loss rate through acid corrosion by 27.43%, and the penetration depth of chloride ions was 2 mm. Xu et al. [37] studied basalt fiber reinforcement for geopolymer concrete. The results showed that the compressive strength and flexural strength of geopolymer concrete increased with the increase in the basalt fiber content, and the basalt fiber could refine the pores. Saloni et al. [38] found that the addition of basalt fibers was beneficial to the fiber–matrix transition zone, and the appropriate content of basalt fibers led to an increase in the setting time and mechanical strength of the concrete. Şahin et al. [39] evaluated the influence of basalt fiber on geopolymer mortars. The results showed that the compressive strength and flexural strength of the fiber-reinforced concrete increased by 25% and 50%, respectively, compared to that of the control samples. Yu et al. [40] investigated the effect of basalt fiber powder on the mechanical properties and microstructure of concrete. The results showed that 0.20 vol% basalt fiber could improve the antiabrasion property significantly, and the incorporation of basalt fiber would refine the pores and promote the densification of the concrete. Wang et al. [41] used MIMICS software to reconstruct 3D CT images of pervious concrete, and then the effect of the fiber length on the mechanical property of pervious concrete was studied. The results showed that the numerical simulation could better reflect the failure process of the specimens. Although many investigations have been carried out on basalt fiber-reinforced concrete, there are few works on the effect of acidic corrosion on the mechanical properties of pervious concrete, and SEM images cannot obtain the distribution of cement paste and aggregate. Therefore, the influence of acidic corrosion and the proportion of basalt fiber on the mechanical property of pervious concrete was studied in this paper, and ICT was used to analyze the internal structure of fiber-reinforced pervious concrete.

**Figure 1:**
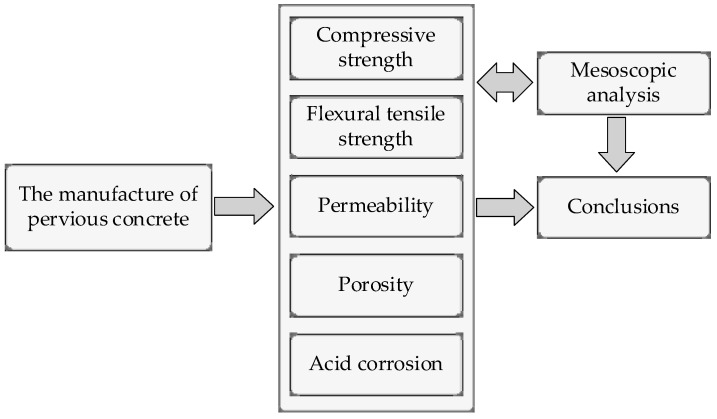
The flow chart of methodology.

In this paper, we further investigated the effect of basalt fiber with different levels of content (2 kg/m^3^, 4 kg/m^3^, 6 kg/m^3^, and 8 kg/m^3^) on the mechanical and physical performance of pervious concrete. This paper is an attempt to establish a balance between the strength and permeability of pervious concrete. The cubic compressive strength, axial compressive strength, flexural tensile strength, permeability, porosity, acidic corrosion behavior, and mesostructure of pervious concrete were studied. The compressive strength and flexural tensile strength increased and then decreased with higher amounts of basalt fiber, while the porosity and permeability decreased as the basalt fiber content increased. Therefore, basalt fibers could achieve enhanced improvement in the physical and mechanical performance of pervious concrete. The research methodology is given in Figure 1.

## 2. Materials and Methods

### 2.1. Materials

#### 2.1.1. Cement

In order to ensure the mechanical property of the pervious concrete, the strength grade of the Portland cement was established as 42.5. This study used the ordinary Portland cement purchased from Shenzhen Yuanheng Building Materials Co., Ltd. (Shenzhen, China), and the performance index provided by this company is given in Table 1.

#### 2.1.2. Silica Fume

Silicon fume is collected and treated from the smoke and dust escaping from the waste gas in the process of melting industrial silicon and ferrosilicon at high temperature in an electric furnace. Adding an appropriate amount of silica fume can improve the fluidity, compactness, and strength of pervious concrete. The silica fume was purchased from Henan Yuanheng Environmental Protection Engineering Co., Ltd. (Zhengzhou, China), and the technical indexes given by the company are presented in Table 2.

#### 2.1.3. Aggregate

The performance of pervious concrete is affected by the type, size, and porosity of the aggregate. In this paper, limestone was selected as the aggregate. The physical properties and grain size distribution of the aggregate were investigated, according to Chinese Code GB/T 14685-2011 [42], as presented in Table 3 and Table 4.

#### 2.1.4. Basalt Fiber

Basalt fiber could improve the mechanical and physical performance of concrete. The basalt fibers used in this paper were provided by Zhejiang Jinshi Co., Ltd. (Hangzhou, China), and the material characteristics were tested and provided by the company, as shown in Table 5.

#### 2.1.5. Other Materials

Polycarboxylate high-performance water reducer is used to reduce the water consumption in the preparation of pervious concrete, so as to improve the fluidity of the concrete. The Q8081PCA liquid equilibrium polycarboxylate acid high-performance water reducer was used in this paper, and the dosage of the water reducer was determined by the trial matching of the specimen. The water used in the pervious concrete was running water.

### 2.2. Mixture Proportion

The impact of material additives was studied by adding basalt fiber into the concrete mix at the levels of 2 kg/m^3^, 4 kg/m^3^, 6 kg/m^3^, and 8 kg/m^3^. The detailed mix design for the experiments is given in Table 6.

### 2.3. Test Method

The pervious concrete was designed to meet the requirements of Chinese Code CJJ/T 135-2009 [43]. The cubic compressive strength, axial compressive strength, and flexural tensile strength of the specimens were tested, according to Chinese Code GB/T 50081-2019 [44]. The testing of the porosity and permeability coefficient was based on Chinese Code CJJ/T 253-2016 [45] and Chinese Code CJJ/T 135-2009 [43]. The acidic corrosion behavior was selected to study the durability of the pervious concrete, according to Chinese Code GB/T 50476-2019 [46]. Three specimens were prepared to determine the individual mechanical and physical performance by average values. All the testing was conducted after the specimens had been cured in a concrete standard curing box for 28 days, with the condition of 22 °C and 95% humidity.

## 3. Experiment and Discussion

### 3.1. Mechanical Properties of Fiber-Reinforced Pervious Concrete

To fully investigate the compressive strength of the pervious concrete, two sizes of specimens were designed: (i). 100 mm × 100 mm × 100 mm, (ii). 100 mm × 100 mm × 400 mm. The groups (i) and (ii) were used to obtain the cubic compressive strength, axial compressive strength, and flexural tensile strength of the pervious concrete, respectively. 

#### 3.1.1. Cubic Compressive Strength

**Figure 2:**
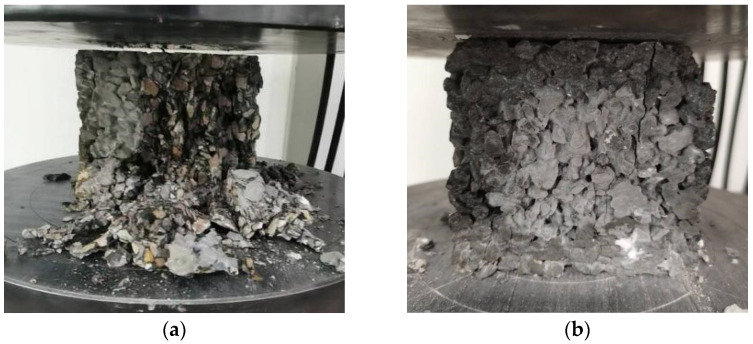
The failure patterns of (**a**) cubic specimens without basalt fiber and (**b**) cubic specimens with basalt fiber.

The failure patterns of the cubic specimens are given in Figure 2. The specimens without basalt fiber were completely destroyed after reaching the maximum bearing capacity, the aggregate was dispersed, and the crushing phenomenon was obvious. Although for the specimens with basalt fiber, some fragments fell off at the corner of the block at the end of testing, but the integrity of the pervious concrete was maintained well. 

**Figure 3:**
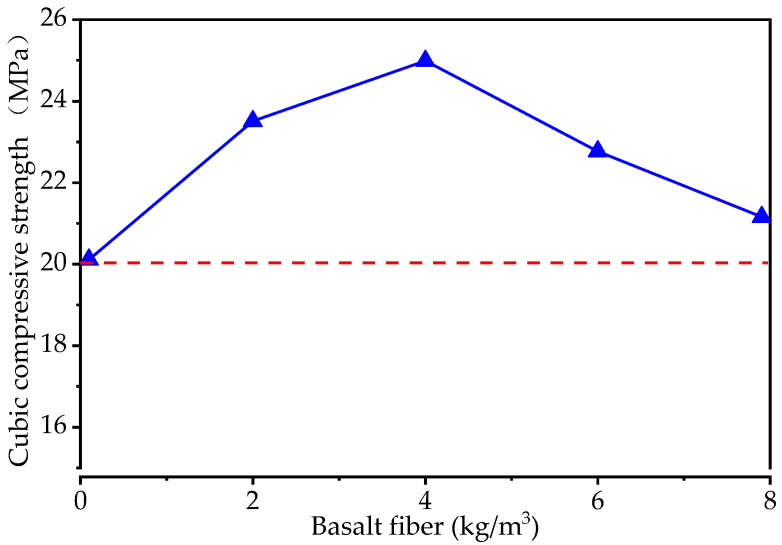
The cubic compressive strength of the specimens with different levels of basalt fiber content.

Figure 3 shows the effect of basalt fiber on the cubic compressive strength of the pervious concrete. Although the cubic compressive strength increases and then decreases as the basalt fiber content increases, the cubic compressive strength value of the pervious concrete with basalt fiber is higher than that of the pervious concrete without basalt fiber (control specimen). The peak value of cubic compressive strength is shown with a basalt fiber content of 4 kg/m^3^, which increases by 24% compared to the control specimen. The red dashed line in Figure 3 indicates that the compressive strength of the pervious concrete can meet the requirements of CJJ/T 135-2009 [43] (20 MPa, the red dashed line in Figure 3).

#### 3.1.2. Axial Compressive Strength

The compressive strength of concrete is related to the shape of the specimen. The prism specimen can better reflect the actual compressive strength of concrete than that of cube samples. Therefore, the axial compressive strength of the pervious concrete was tested in this paper. 

**Figure 4:**
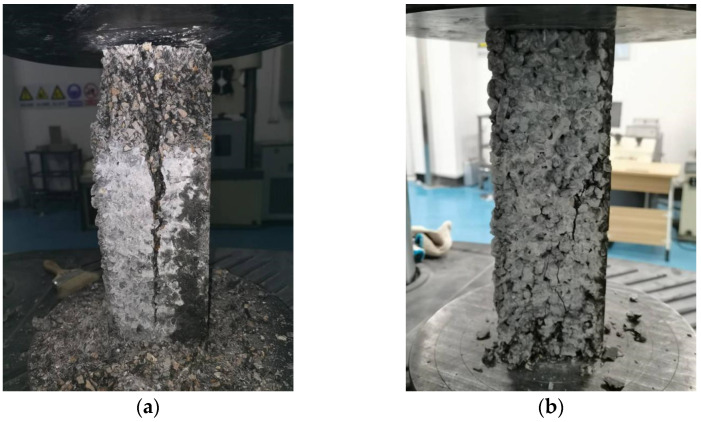
The failure patterns of (**a**) concrete prism without basalt fiber and (**b**) concrete prism with basalt fiber under axial compression.

The failure patterns of concrete prisms under the action of axial load are given in Figure 4. For the specimens without basalt fiber, a wide vertical crack appeared in the middle of the prism. Although the prism was not completely destroyed, it had completely lost the bearing capacity. Although for the specimen with basalt fiber, small cracks occurred on the side of the specimen and a small amount of the aggregate fell off, the specimen as a whole remained intact. 

**Figure 5:**
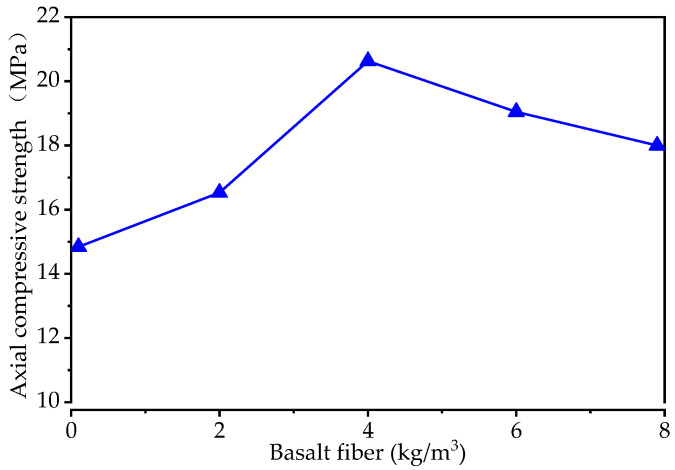
The axial compressive strength of specimens with different levels of basalt fiber content.

The effect of basalt fiber on the axial compressive strength of the pervious concrete is presented in Figure 5. It can be seen that with an increase in the basalt fiber content, there is an increase and then a decrease in the axial compressive strength. The axial compressive strength of the pervious concrete increases from 14.84 MPa for the control specimen (i.e., the basalt fiber content is 0 kg/m^3^) to 20.63 MPa for a basalt fiber content of 4 kg/m^3^, and then decreases to 18.00 MPa for a basalt fiber content of 8 kg/m^3^. The results also indicate that the addition of basalt fiber can improve the axial compressive strength of pervious concrete.

#### 3.1.3. Flexural Tensile Strength

Pervious concrete is inevitably subjected to flexural and tensile loads in the practical usage process. Therefore, it is essential to study the flexural tensile strength to prevent the appearance of cracks in pervious concrete. 

**Figure 6:**
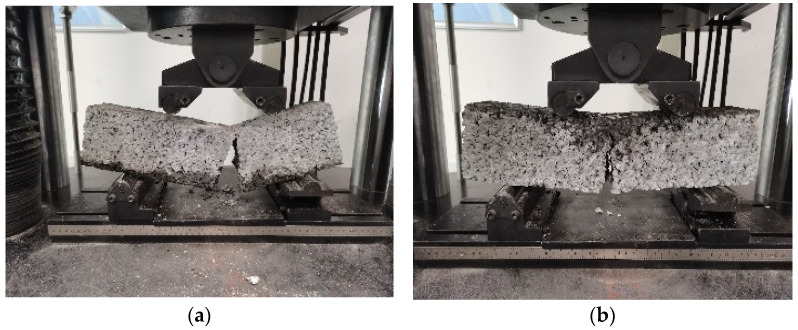
The flexural tensile failure patterns of (**a**) concrete prism without basalt fiber and (**b**) concrete prism with basalt fiber.

Figure 6 shows the failure pattern of a concrete prism under the action of a lateral load. Whether or not the basalt fiber was added to the pervious concrete, the cracks mainly appeared in the middle of the specimens. The difference between the specimens with basalt fiber and control specimens is that without the connection of the fiber, once the crack appeared at the bottom, it would develop rapidly to the top of the specimen.

**Figure 7:**
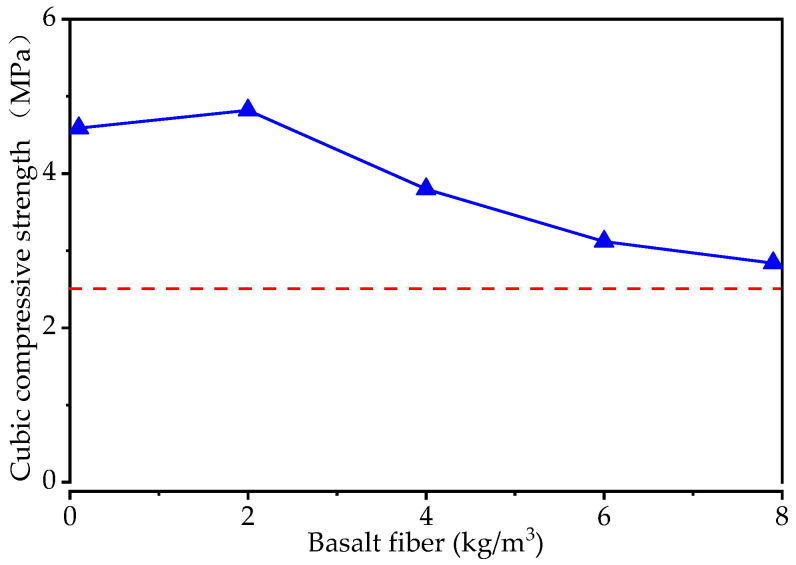
The flexural tensile strength of the specimens with different levels of basalt fiber content.

The effect of basalt fiber on the flexural tensile strength of the pervious concrete is presented in Figure 7. The flexural tensile strength increases and then decreases with the increasing of the basalt fiber content. The maximum strength value is 4.82 MPa when the basalt fiber content is 2 kg/m^3^, and then the strength value decreases to 2.84 MPa for the basalt fiber content of 8 kg/m^3^. In CJJ/T 135-2009 [43], it is stated that the flexural tensile strength should not be lower than 2.5 MPa (the red dashed line in Figure 7), meaning that all the specimens met the application requirement.

Combining the results of the cubic compressive strength, axial compressive strength, and flexural tensile strength, it could be concluded that the mechanical properties of the fiber-reinforced pervious concrete can meet the requirements of CJJ/T 135-2009 [43], and that the appropriate addition of basalt fiber could improve the mechanical properties.

### 3.2. Permeability and Porosity of Fiber-Reinforced Pervious Concrete

#### 3.2.1. Permeability

**Figure 8:**
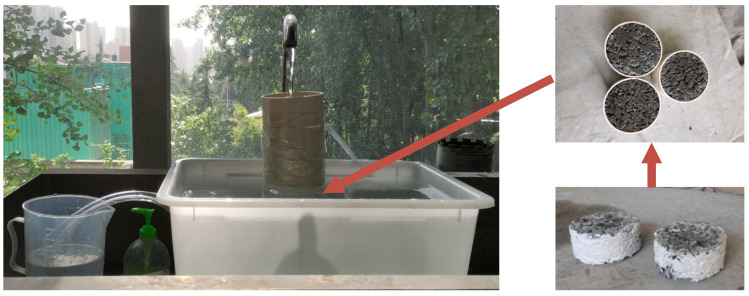
The testing of permeability coefficient.

**Figure 9:**
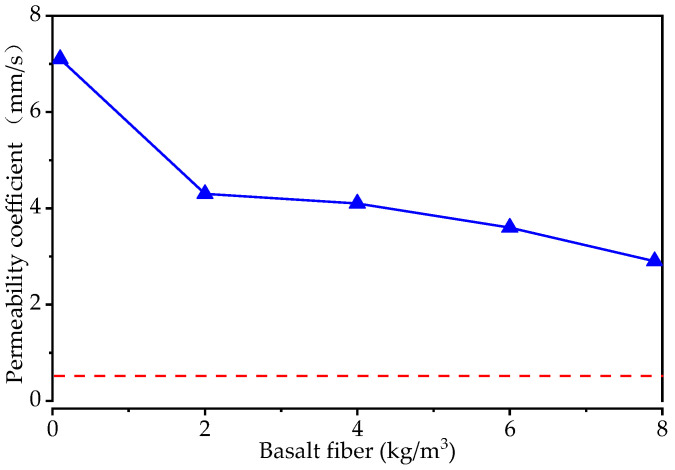
The permeability coefficient of pervious concrete with different levels of basalt fiber content.

Cylinder specimens were prepared to obtain the permeability coefficient, as given in Figure 8. The cylinder specimen should be connected to the cylinder wall using gasket cement, to ensure that the water can only flow through the specimen. The permeability coefficient could be obtained from Equation (1).

(1) $$k_{T}=\frac{QL}{AHt}$$

in which $k_{T}$ is the permeability coefficient when the water temperature is *T* °C, *Q* is the amount of water seeping out within *t* seconds, *L* is the thickness of the specimen, *A* is the upper surface area of the specimen, *H* is the water level difference, and *t* is the time. The results of the calculation are shown in Figure 9.

As shown in Figure 9, the permeability coefficient (mm/s) decreases as the basalt fiber content increases. Compared to the specimen without basalt fiber, the permeability coefficient decreases rapidly (0–2 kg/m^3^) and then slowly (2–8 kg/m^3^) with the increasing of the basalt fiber content. As can be seen, the permeability coefficient is roughly 2.9–7.1 mm/s, which is higher than the regulation of CJJ/T 135-2009 [43] (0.5 mm/s, the red dashed line in Figure 9).

#### 3.2.2. Porosity

**Figure 10:**
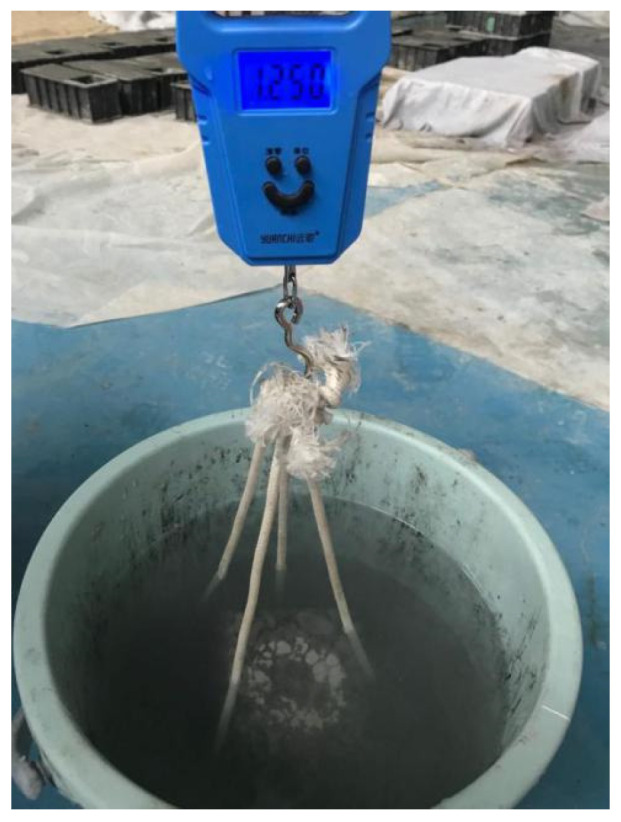
The testing of porosity.

As shown in Figure 10, the cubic specimens were immersed in water for one day, and then the specimens were taken out to be dried until the surfaces were dried. The porosity could be obtained through the quality difference before and after drying. The porosity can be calculated by Equation (2).

(2) $$P=\left[1-\left(\frac{m_{2}-m_{1}}{\rho_{W}V_{0}}\right)\right]\times 100\%$$

in which P is porosity, $m_{1}$ is the quality of the specimen under water, $m_{2}$ is the quality of the specimen after air drying for 24 h, $\rho_{W}$ is the density of water at normal temperature, and $V_{0}$ is the volume of the specimen.

**Figure 11:**
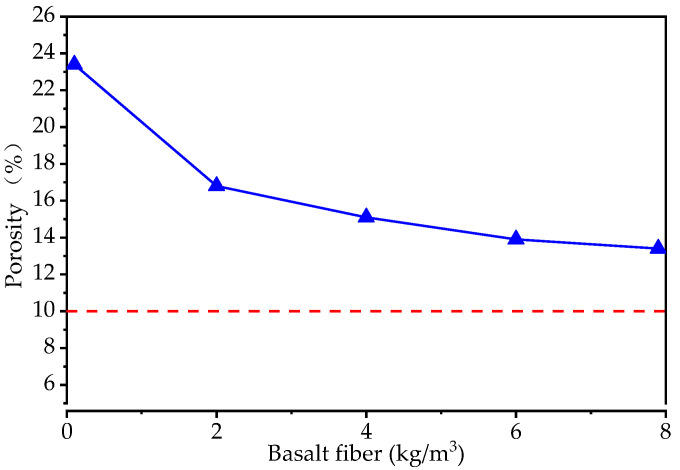
The porosity of the specimens with different levels of basalt fiber content.

The porosity of the specimens is given in Figure 11. The porosity of the specimens, when compared with the control specimens, showed a percentage decrease of 28.2%, 35.5%, 40.6%, and 42.7% for the basalt fiber contents of 2 kg/m^3^, 4 kg/m^3^, 6 kg/m^3^, and 8 kg/m^3^, respectively. However, the porosity could meet the requirements of CJJ/T 135-2009 [43] (10%, the red dashed line in Figure 11). It is clear that the porosity has a relationship with the permeability, and the addition of basalt fibers did not change this trend. 

#### 3.2.3. The Relationship between the Permeability and Porosity

**Figure 12:**
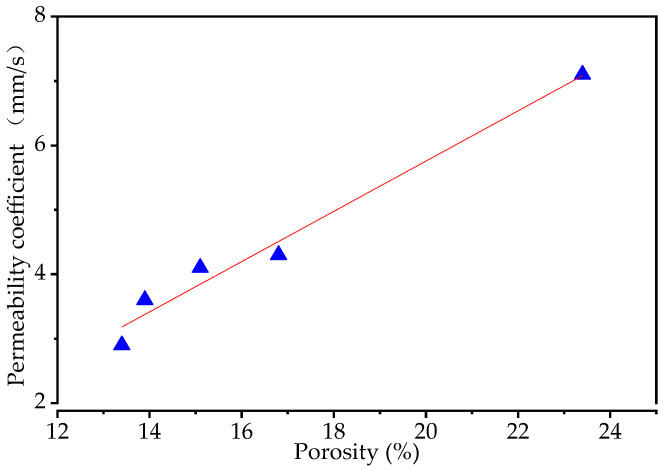
The relationship between permeability and porosity.

It is well known that the porosity is vital to the permeability. Therefore, this paper presented the relationship between the permeability coefficient and porosity in Figure 12, in which the red line (fitting curve) is the result of the regression analysis. The formula of the fitting curve can be expressed as Equation (3).

(3) $$k_{T}=-2.04+0.39P$$

It can be seen from Figure 12 that the permeability coefficient is linearly dependent on porosity, and the permeability coefficient increases with the increasing of porosity. 

### 3.3. The Acidic Corrosion Behavior of Fiber-Reinforced Pervious Concrete

**Figure 13:**
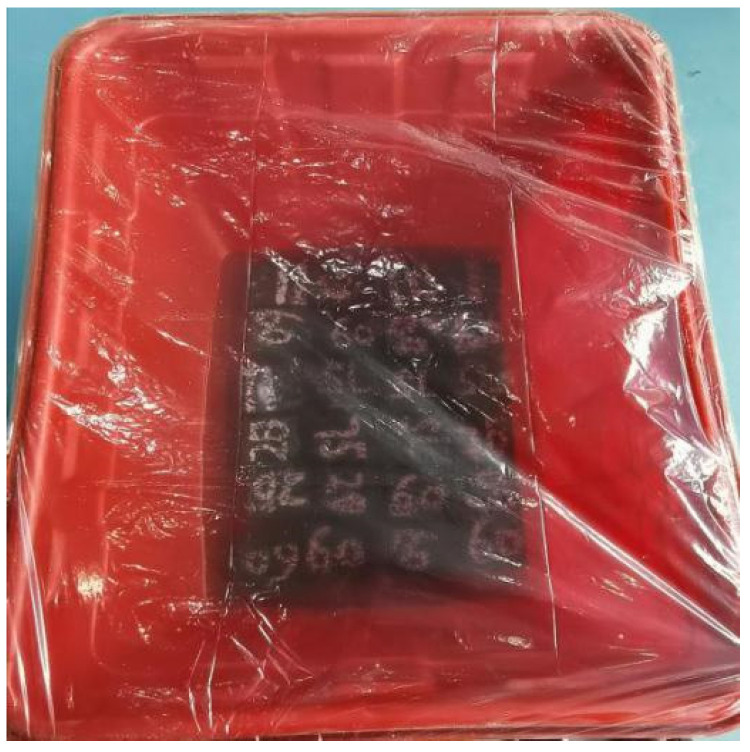
The acidic corrosion of the specimens.

Due to the large number of pores, corrosive substances can easily enter the interior of pervious concrete, which will destroy the internal structure of pervious concrete and reduce the performance of pervious concrete. In this paper, the specimens were put into acid solution (hydrochloric acid solution with pH = 1), whose surface was at least 20 mm higher than the upper surface of the specimens, as shown in Figure 13. The specimens were soaked in the acid solution for 20 days, 40 days, and 60 days.

**Figure 14:**
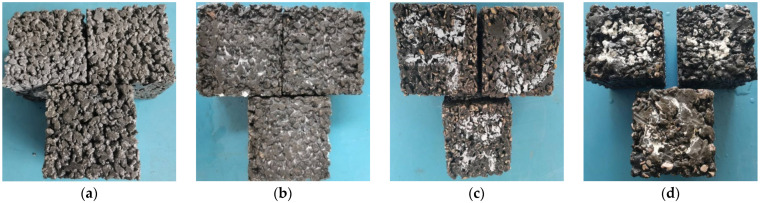
The testing phenomenon of specimens (with basalt fiber content of 4 kg/m^3^) for acidic corrosion: (**a**) 0 day, (**b**) 20 days, (**c**) 40 days, and (**d**) 60 days.

The corrosion phenomenon of the specimens (with a basalt fiber content of 2 kg/m^3^) is shown in Figure 14, and the specimens with the other levels of basalt fiber content exhibited the same experimental phenomenon. It could be seen that when the corrosion time was 20 days, the aggregate did not fall off, and a white colloid appeared, almost filling the surface gap of the specimen; when the corrosion time lasted for 40 days, the aggregate was obviously exposed and fell off slightly, the white colloid gradually disappeared, and the soaking solution became turbid; when the corrosion time was 60 days, the surface of the specimens was seriously corroded and became dark brown, and the specimens showed an uneven shape due to the aggregate falling off (at the corners of the specimens).

**Figure 15:**
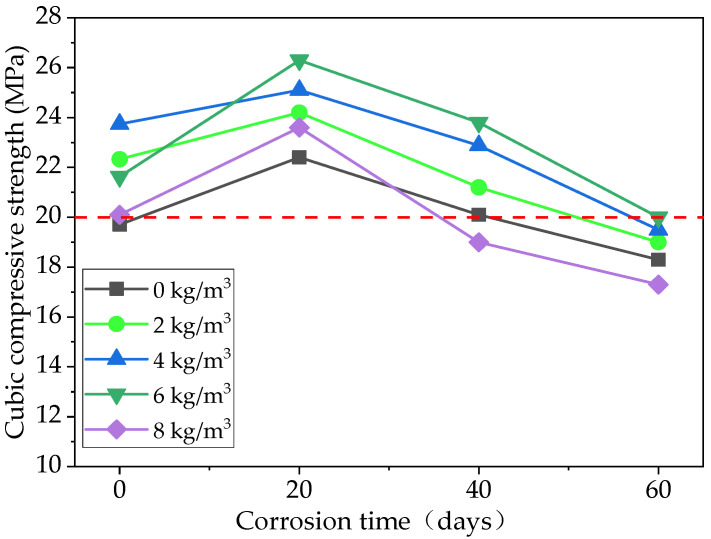
The cubic compressive strength of specimens with different levels of basalt fiber content and different corrosion times.

Figure 15 shows the effect of the basalt fiber content and acidic corrosion time on the cubic compressive strength of the specimens. As shown in Figure 15, for the pervious concrete specimen, which had a different level of basalt fiber content, the cubic compressive strength increases and then decreases as the acidic corrosion time increases. The reason for increasing the compressive strength is the production of acidic corrosion. CaSO_4_·2H_2_O, CaAl_2_Si_2_O_8_, and Ca_3_Al_6_O_12_·CaSO_4_ are produced due to acidic corrosion. When the corrosion time is short, these expansive substances could block the pores and reduce the corrosion speed of the pervious concrete. However, for the acidic corrosion of 40 days and 60 days, the expansive substance gradually decomposes in the acid solution, which can accelerate the corrosion process of the pervious concrete [47]. It can also be seen from Figure 15 that the cubic compressive strength cannot meet the requirements of CJJ/T 135-2009 [43] with acidic corrosion of 60 days, indicating that the acidic corrosion will seriously affect the mechanical properties of pervious concrete. 

### 3.4. The Mesostructure of Fiber-Reinforced Pervious Concrete

**Figure 16:**
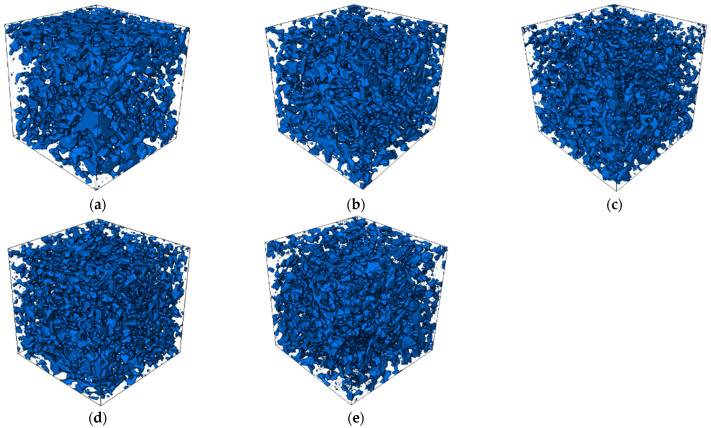
The 3D images of the specimens with different levels of content of basalt fiber: (**a**) 0 kg/m^3^, (**b**) 2 kg/m^3^, (**c**) 4 kg/m^3^, (**d**) 6 kg/m^3^, and (**e**) 8 kg/m^3^.

**Figure 17:**
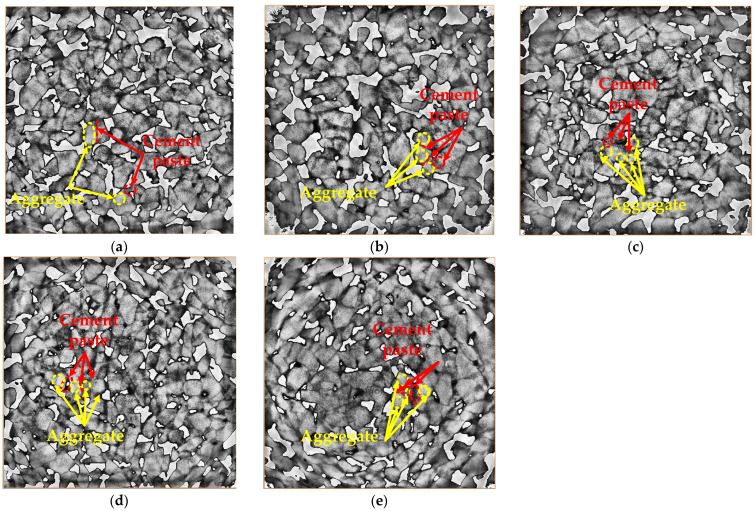
The 2D images of cross sections of the specimens with different levels of content of basalt fiber: (**a**) 0 kg/m^3^, (**b**) 2 kg/m^3^, (**c**) 4 kg/m^3^, (**d**) 6 kg/m^3^, and (**e**) 8 kg/m^3^.

In order to obtain the mesostructure of the pervious concrete, industrial computed tomography (ICT) was used to scan the specimen. Figure 16 and Figure 17, which can be obtained after processing with Avizo software, are the 3D images and 2D images of the cross section respectively. The blue-scale in Figure 16 and the grey-scale in Figure 17 are the aggregates and the cement paste. As shown in Figure 16, the proper addition of basalt fiber is beneficial to uniform dispersion of aggregate and cement paste (Figure 16a–c), while too much basalt fiber causes more cement paste to bond to the fiber, meaning that the bond between the aggregates and the cement paste will be weakened due to the reduction in cement paste on the aggregates (Figure 16d,e). 

The same trend can be seen from Figure 17. For the control specimen (Figure 17a), the cement paste is mainly bonding to the aggregate, and there are lots of pores in the pervious concrete. With the increase in the basalt fiber content, more and more cement paste is bonded to the fiber, which reduces the pores in the pervious concrete (Figure 17b–e). 

### 3.5. Analysis of the Relation between Mechanical Properties and Physical Performance

**Figure 18:**
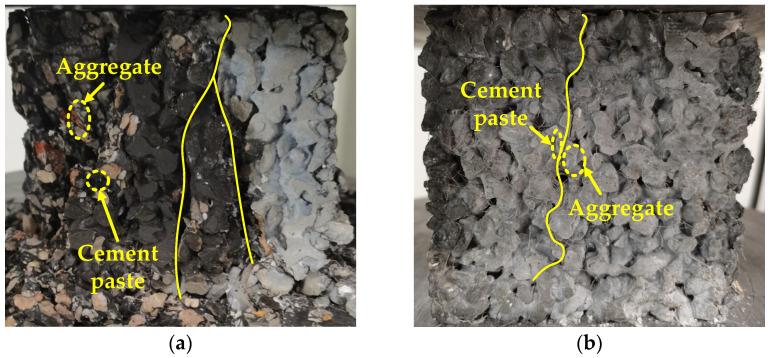
The failure of the specimen: (**a**) sample without basalt fiber and (**b**) sample with basalt fiber.

As described in [48], pervious concrete could be divided into three parts: aggregate, cement paste, and the interfacial transition zone (ITZ), in which the ITZ represents the point/path at which there is debonding between the aggregates and the cement paste. The same phenomenon can also be seen in Figure 16 and Figure 17. Therefore, three different failure modes could be observed—aggregate failure, cement paste failure, and ITZ failure. The failures are given in Figure 18 with the yellow marks. In the testing of cubic compressive strength, for the specimens without or with less basalt fiber content (0 kg/m^3^, 2 kg/m^3^, and 4 kg/m^3^), three failure modes could be observed, while for the specimens with a higher basalt fiber content (6 kg/m^3^ and 8 kg/m^3^), the failure was mainly along the ITZ. The same phenomenon could also be seen in the testing of axial compressive strength. Considering the changes in the strength value of mechanical performance, it could be concluded that the proper addition of basalt fiber helps to achieve the strength balance of the aggregate, cement paste, and the ITZ, which will ensure the strength of the specimen. Although excessive basalt fiber results in poor characteristics of the ITZ and inferior strength properties. 

It is widely accepted that porosity is vital to the mechanical performance and physical properties of pervious concrete. As described in the testing results of permeability and porosity, and taking the mesostructure of the fiber-reinforced pervious concrete into account, the addition of basalt fiber causes more cement to bond to the fibers, which blocks the pores in pervious concrete. It is also clear that porosity has a positive relationship with permeability, and the addition of basalt fibers does not change this trend. 

In order to improve the physical and mechanical properties of pervious concrete and promote the application of pervious concrete in actual construction, the optimum basalt fiber content should be established. Comparing the samples with a basalt fiber content of 0 kg/m^3^ (control samples) and 4 kg/m^3^, the cubic compressive strength and axial compressive strength increased by 24% and 39%, respectively, while the flexural tensile strength, permeability, and porosity were reduced by 17%, 42%, and 35%, respectively, with the 4 kg/m^3^ basalt fiber content. For the specimens for acidic corrosion of 0 day, 20 days, 40 days, and 60 days, the cubic compressive strength of the samples with a basalt fiber content of 4 kg/m^3^ was 21%, 12%, 14%, and 7% higher than that of the control specimens, respectively. All the testing results meet the requirements of specification, indicating that the optimum basalt fiber content should be determined as 4 kg/m^3^.

## 4. Conclusions

This study investigated the effect of basalt fiber on the physical and mechanical properties of pervious concrete. To achieve the aim of this paper, the pervious concrete matrix was mixed incorporating basalt fiber at the level of 2 kg/m^3^, 4 kg/m^3^, 6 kg/m^3^, and 8 kg/m^3^. The strength testing, porosity testing, permeability testing, and acid corrosion testing were conducted on the testing specimen. The following conclusions were made:

1. The incorporation of basalt fiber enhances the cubic and axial compressive strength, and the compressive strength increases and then decreases with the increasing of the basalt fiber content. Compared to the matrix without basalt fiber, the cubic and axial compressive strength of the matrix with a basalt content of 4 kg/m^3^ increased by 24% and 39%, respectively.
2. The contribution of basalt fiber to the flexural strength of the concrete matrix reduces with the increasing of the basalt fiber content. Compared to the control samples, the flexural tensile strength of the matrix with a basalt fiber content of 4 kg/m^3^ reduces by 17%.
3. A pervious concrete matrix incorporating basalt fiber showed a lower permeability and porosity than that of the mix without fiber. Moreover, the permeability coefficient and the porosity of the pervious concrete with a basalt fiber content of 4 kg/m^3^ were 42% and 35% lower than those of the control samples.
4. For the concrete matrix with the basalt fiber content of 4 kg/m^3^, the cubic compressive strengths of the matrix with acidic corrosion of 0 day, 20 days, 40 days, and 60 days were 21%, 12%, 14%, and 7% higher than those of the control group, respectively. The compressive strength increased and then decreased with the increase in the corrosion time, and the addition of basalt fibers does not change this trend.
5. The testing results of ICT scanning indicated that the basalt fiber obviously affects the distribution of the cement paste and aggregate, hence affecting the physical and mechanical properties of the pervious concrete. The optimum basalt fiber content can realize the balance between the cement paste, aggregate, and the ITZ.

The physical and mechanical properties of fiber-reinforced pervious concrete could satisfy the requirements of CJJ/T 135-2009. The obtained results indicated that it is possible to investigate whether pervious concrete is suitable for structural application by the addition of basalt fiber, which can maximize the application of pervious concrete.

## Figures and Tables

**Table 1:** The performance indexes of cement.

Initial Setting Time (min)	Final Setting Time (min)	Flexural Strength (MPa)		Compressive Strength (MPa)	
		3 d	28 d	3 d	28 d
255	290	5.2	8.85	24.6	48.4

**Table 2:** The main technical indexes of silica fume.

Fire-Resistance Temperature (°C)	Volumetric Weight (kg/m^3^)	Mean Diameter (µm)	Specific Surface Area (m^2^)
1600	1650	0.2	24

**Table 3:** Physical properties of aggregate.

Aggregate Size (mm)	Bulk Density (kg/m^3^)	Apparent Density (kg/m^3^)	Porosity (%)
5–10	1580	2750	40.2

**Table 4:** Sieve analysis of aggregate.

Sieve Size (mm)	2.36	4.75	9.5
Accumulated retained percentage (%)	99	95	11

**Table 5:** The technical indexes of basalt fiber.

Density (kg/m^3^)	Tensile Strength (MPa)	Elastic Modulus (GPa)	Length (mm)	Diameter (µm)	Elongation (%)
2650	4800	110	20	15	3.1

**Table 6:** Mix proportion of the pervious concrete.

Number	Material Consumption (kg/m^3^)					
	Aggregate	Cement	Water	Silica Fume	Water-Reducing Agent	Basalt Fiber
BF-0	1617	388	121	25	4.13	0
BF-2	1617	388	121	25	4.13	2
BF-4	1617	388	121	25	4.13	4
BF-6	1617	388	121	25	4.13	6
BF-8	1617	388	121	25	4.13	8

## Data Availability

Data sharing is not applicable to this article.
